# Cooperative function of synaptophysin and synapsin in the generation of synaptic vesicle-like clusters in non-neuronal cells

**DOI:** 10.1038/s41467-020-20462-z

**Published:** 2021-01-11

**Authors:** Daehun Park, Yumei Wu, Sang-Eun Lee, Goeun Kim, Seonyoung Jeong, Dragomir Milovanovic, Pietro De Camilli, Sunghoe Chang

**Affiliations:** 1 Departments of Neuroscience and Cell Biology, Howard Hughes Medical Institute, Program in Cellular Neuroscience, Neurodegeneration and Repair, Kavli Institute for Neuroscience, Yale University School of Medicine, New Haven, CT 06510 USA; 2 Department of Physiology and Biomedical Sciences, Seoul National University College of Medicine, Seoul, 03080 South Korea; 3 Laboratory of Molecular Neuroscience, German Center for Neurodegenerative Diseases (DZNE), Charitéplatz 1, 10117 Berlin, Germany

**Keywords:** Intrinsically disordered proteins, Supramolecular assembly, Cellular neuroscience, Synaptic vesicle exocytosis

## Abstract

Clusters of tightly packed synaptic vesicles (SVs) are a defining feature of nerve terminals. While SVs are mobile within the clusters, the clusters have no boundaries consistent with a liquid phase. We previously found that purified synapsin, a peripheral SV protein, can assemble into liquid condensates and trap liposomes into them. How this finding relates to the physiological formation of SV clusters in living cells remains unclear. Here, we report that synapsin alone, when expressed in fibroblasts, has a diffuse cytosolic distribution. However, when expressed together with synaptophysin, an integral SV membrane protein previously shown to be localized on small synaptic-like microvesicles when expressed in non-neuronal cells, is sufficient to organize such vesicles in clusters highly reminiscent of SV clusters and with liquid-like properties. This minimal reconstitution system can be a powerful model to gain mechanistic insight into the assembly of structures which are of fundamental importance in synaptic transmission.

## Abstract (web summary)

Synaptic vesicle clusters were proposed to represent phase separated condensates. Here, the authors show that only two proteins, synapsin and synaptophysin, are sufficient to make vesicle clusters in fibroblasts which are similar to those found at synapses in morphology and liquid-like properties.

## Introduction

A characteristic feature of presynaptic nerve terminals is the presence of tight clusters of synaptic vesicles (SVs) anchored to active zones of secretion of the presynaptic plasma membrane. As vesicles are consumed by fusion with this membrane, new vesicles generated by endocytic membrane recycling join the clusters and intermix with pre-existing vesicles, suggesting a fluid nature of the clusters^1^. It was recently suggested that principles of liquid-liquid phase separation (LLPS) may explain the formation and maintenance of these clusters^1^. LLPS is the process in which particles present in a liquid medium coalesce into a distinct liquid phase in spite of the absence of a retaining boundary^2–4^. Synapsin (Syn), a dimeric SV-associated protein, was proposed to be a key player in the formation of these condensates^5–7^. Purified synapsin can phase separate either alone, or via interactions with presynaptically enriched binding partners^7^. Synapsin also binds acidic lipid bilayers^8^ and, when incubated with small artificial vesicles, can trap them into its phase^7,8^. Lack of synapsin function, either due to genetic disruption or to antibody microinjection, impairs clustering of SVs at synapses with the exception of the pool of vesicles most close to the presynaptic plasma membrane^9–12^. Until now, however, whether synapsin is sufficient to organize vesicles in living cells and how it cooperates with membrane proteins in this process is not known. To begin address these questions we explored the possibility of reconstituting a SV cluster in an ectopic location, the cytoplasm of a non-neuronal cell.

We had previously shown that expression in fibroblastic CHO cells of synaptophysin (Syph), a major SV intrinsic membrane protein, results in its accumulation in synaptic-like microvesicles which, like bona fide SVs, are part of the recycling endocytic system, as they become labeled by endocytic tracers^13,14^. These vesicles are often arranged in very small clusters^13^, but not in the larger tightly packed clusters typical of SVs in presynaptic nerve terminals^1^. We have now investigated a potential synergistic effect of synapsin and synaptophysin in generating structures similar to those found in nerve terminals. Our results show that expression of these two neuronal proteins alone in fibroblasts is sufficient to generate clusters of small vesicles that are morphologically similar to those typical of synapses and that share some of the same properties.

## Results

### Synapsin and synaptophysin-positive vesicles coalesce into a distinct phase in fibroblastic cells expressing both proteins

**Fig. 1:**
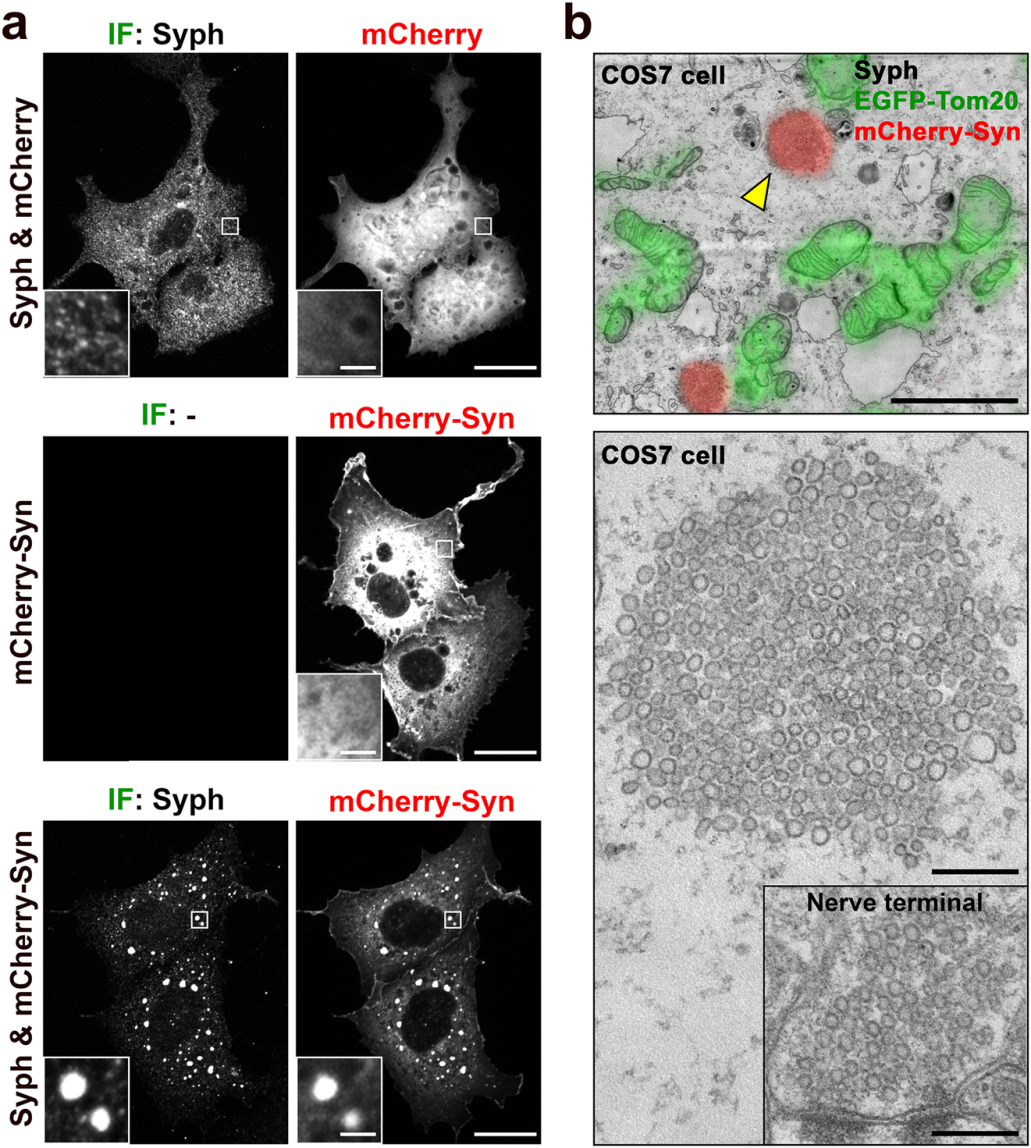
Co-expression of synaptophysin with mCherry-synapsin in non-neuronal cells results in droplets represented by clusters of small vesicles resembling SVs. **a** COS7 cells were transfected as indicated and synaptophysin was detected by immunofluorescence (IF). Insets shows small regions at higher magnification. **b** Correlative light electron microscopy (CLEM) of COS7 cells expressing synaptophysin, mCherry-synapsin and EGFP-TOM20. Top: superimposition of fluorescence and EM. The arrowhead point to the spot whose EM appearance is shown at higher magnification at the bottom. Inset: a synaptic vesicle cluster in the nerve terminal of a mouse brain section is shown at the same magnification as a comparison. Scale bars, **a** = 20 μm (2 μm for insets), **b** = 2 μm (top) or 200 nm (bottom and inset).

**Fig. 2:**
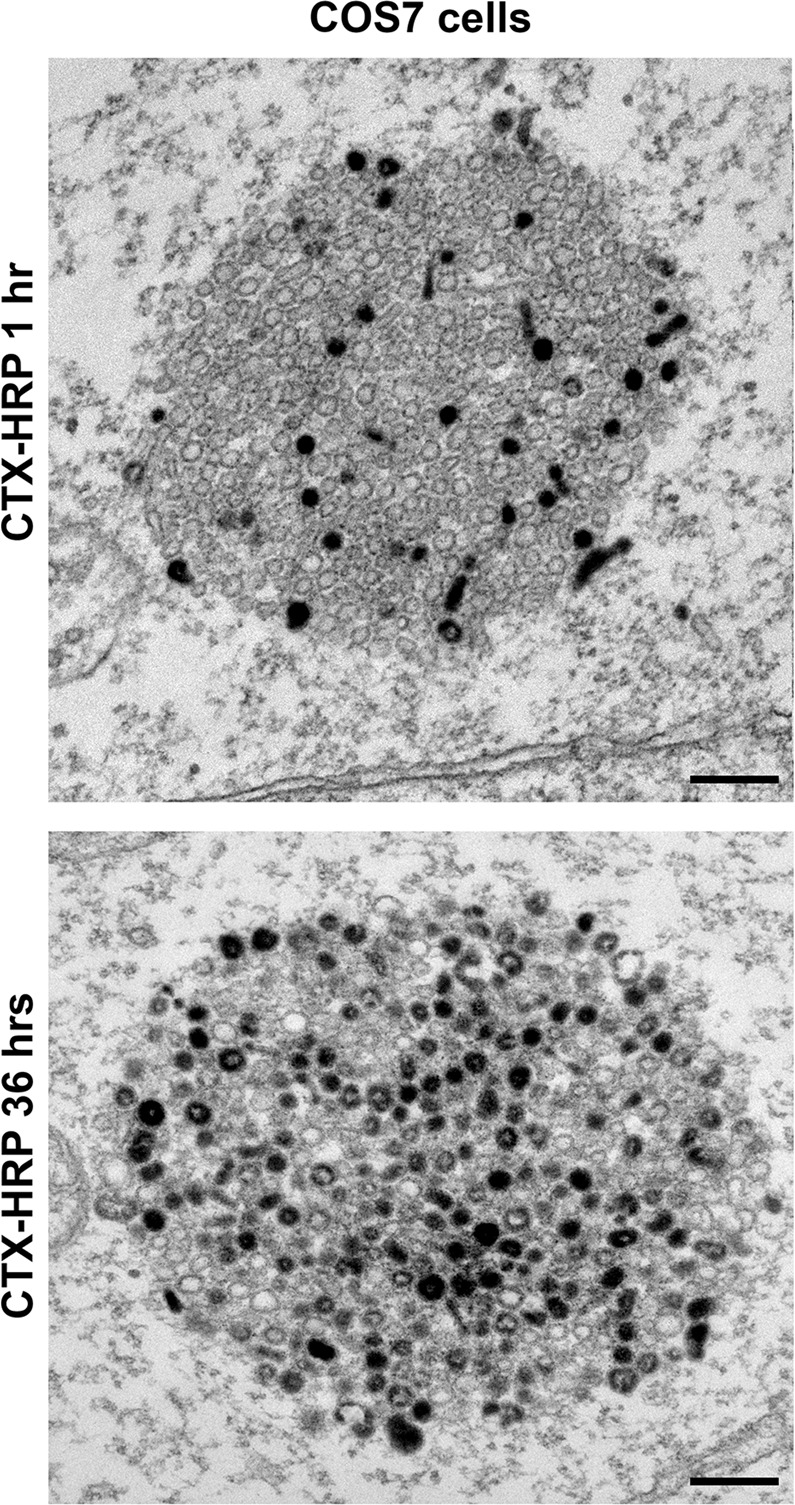
Vesicles labeled by an endocytic tracer are randomly intermixed with unlabeled vesicles in vesicle clusters. Synaptophysin and mCherry-synapsin expressing COS7 cells were treated with 10 μg/ml cholera toxin conjugated HRP (CTX-HRP) for either 1 h or 36 h and then fixed for electron microscopy. Vesicles labeled by CTX-HRP are randomly interspersed with unlabeled vesicles within the clusters. Scale bars = 200 nm.

Anti-synaptophysin immunofluorescence of COS7 cells transfected with untagged synaptophysin (Syph) revealed a fine punctate localization (Fig. 1a), which, as described previously^13,14^, reflects small vesicles of the endosomal system often arranged in small clusters. Meanwhile, mCherry-synapsin (mCherry-Syn) or EGFP-synapsin (EGFP-Syn), transfected alone in the same cells, had a diffuse cytosolic distribution (Fig. 1a and Supplementary Fig. 1a,b), in spite of the property of purified synapsin to phase separate when incubated alone^7^. However, when mCherry-synapsin and synaptophysin were co-expressed together, very large droplets positive for both proteins (as assessed by mCherry fluorescence and immunofluorescence (IF), respectively) were present throughout the cytoplasm (Fig. 1a). Correlative light-electron microscopy (CLEM) identified these droplets as collections of small tightly apposed vesicles similar in size to bona fide SVs (Fig. 1b). Furthermore, preincubation of cells before fixation with the endocytic tracer cholera toxin-horseradish peroxidase (CTX-HRP), showed that a fraction of these vesicles were positive for the tracer, and that the percent of vesicles positive for the tracers increased with the time of incubation in the presence of the tracer (Fig. 2). These findings indicate that vesicles within the clusters represent organelles of the endosomal system, as expected for SV-like organelles.

**Fig. 3:**
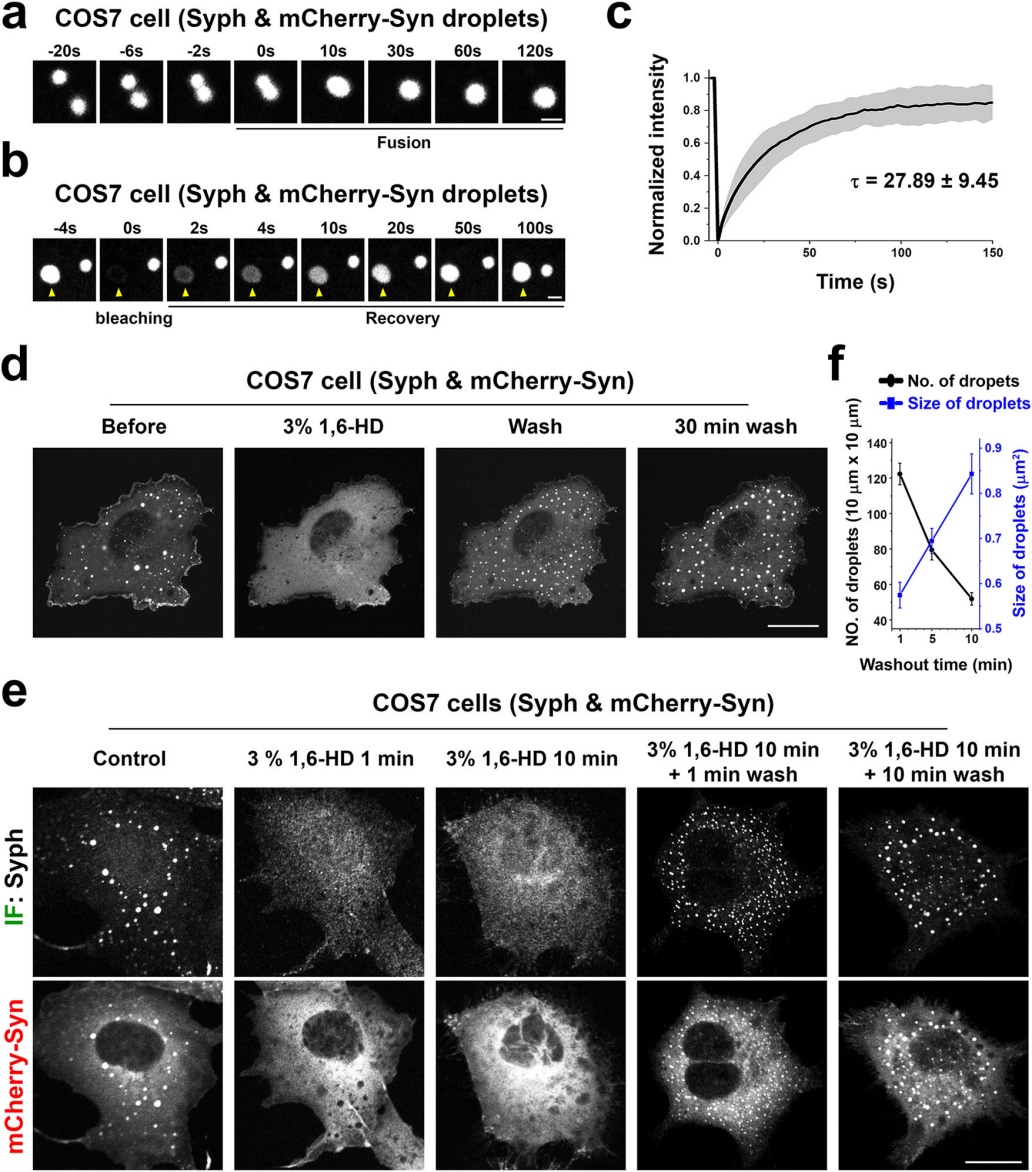
Liquid like behaviors of droplets containing synaptophysin and mCherry-synapsin in living cells. **a**–**f** COS7 cells transfected with synaptophysin and mCherry-synapsin. **a** Fusion of two droplets. **b** Representative time-lapse images showing fluorescence recovery of mCherry-synapsin after photobleaching of a single droplet (arrowhead). **c** Plot of the average fluorescence intensities after photobleaching of multiple spots. Fluorescence recovery traces were fitted to measure the time constant τ of fluorescence recovery kinetics. Data are represented as mean ± SD (*n* = 7). **d** and **e** Droplets disperse reversibly upon 3% 1,6-Hexanediol (1,6-HD) treatment. **d** shows time-lapse images of mCherry-synapsin in the same cell. **e** shows different cells treated as indicated and then fixed for the visualization of synaptophysin by immunofluorescence. **f** Time-dependent changes in the number and average size of droplets after 1,6-Hexanediol washout. *n* = 35 (1 min wash), 41 (5 min wash), and 34 (10 min wash) cells from two independent assays. Values are means ± SEM. Scale bars, **a** = 2 μm, **b** = 2 μm, **d** = 20 μm, or **e** = 20 μm.

**Fig. 4:**
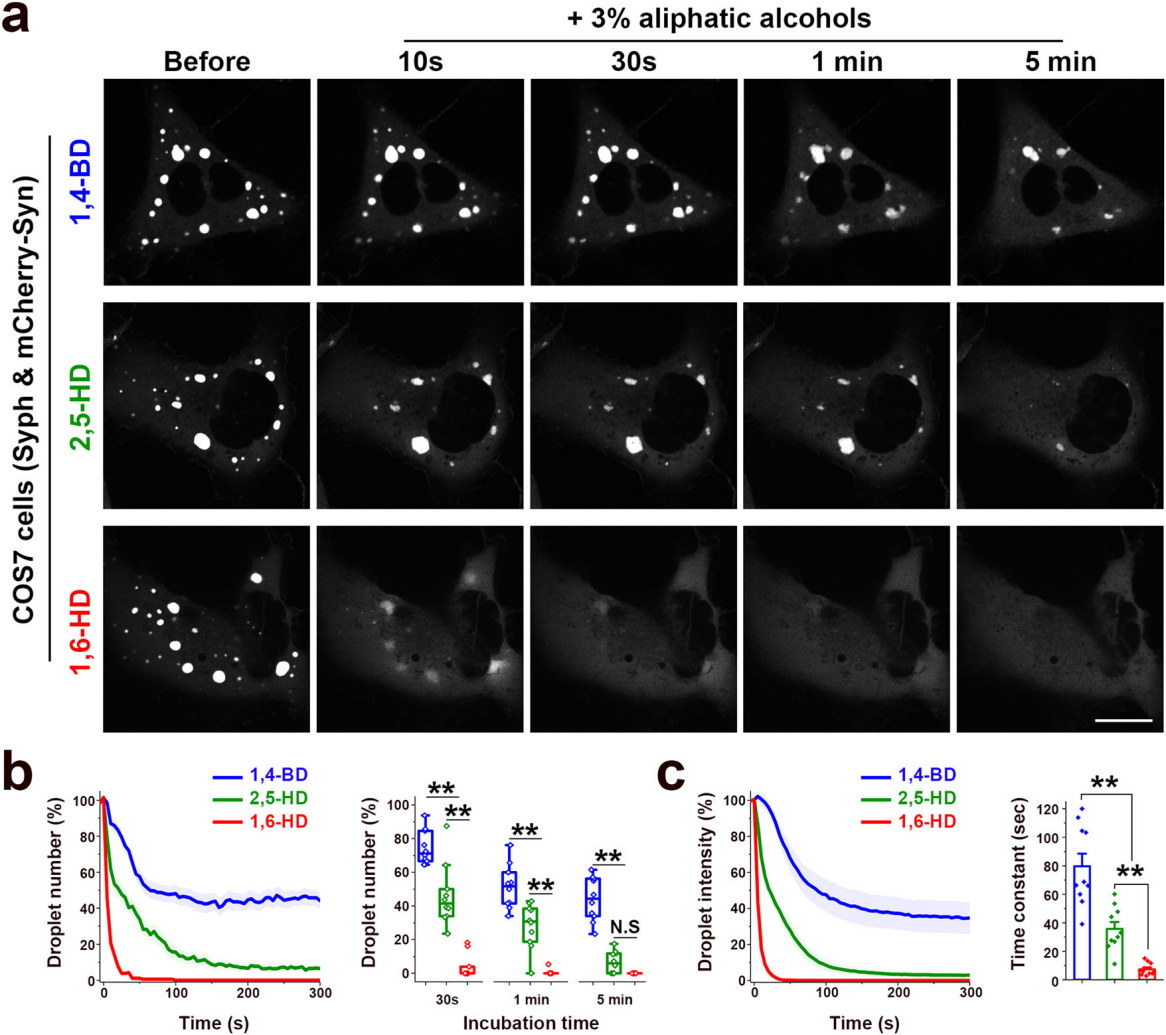
Effects of different aliphatic alcohols on synaptophysin-synapsin droplets. **a**–**c** COS7 cells were transfected with synaptophysin and mCherry-synapsin and then treated with 3% aliphatic alcohols (1,4-BD: 1,4-Butanediol, 2,5-HD: 2,5-Hexanediol, 1,6-HD: 1,6-Hexanediol). **a** Representative time-lapse images. Scale bar = 20 μm. **b**, Left: Time-dependent changes in the number of droplets. Right: Box plots show median line (midline), 25/75 percentiles (boxes), and range (whiskers) at three different time points after treatment. **c**, Analysis of the fluorescence intensities of droplets after addition of aliphatic alcohols. Left: Integrated intensity of droplets at each timepoints were normalized to the initial value (before treatment). Right: The decay time constant (τ) was calculated by fitting the decay trace with a single exponential function. Values are means ± SEM; N.S., not significant; **p < 0.01 by one-way ANOVA and Tukey’s HSD post hoc test. *n* = 10 independent experiments for each groups. *p* values (**b**): 8.09563E-5 (1,4-BD vs. 2,5-HD at 30 s), 0 (1,4-BD vs. 1,6-HD at 30 s), 1.29319E-7 (2,5-HD vs. 1,6-HD at 30 s), 1.53526E-4 (1,4-BD vs. 2,5-HD at 1 min), 0 (1,4-BD vs. 1,6-HD at 1 min), 1.47736E-5 (2,5-HD vs. 1,6-HD at 1 min), 0 (1,4-BD vs. 2,5-HD at 5 min), 0 (1,4-BD vs. 1,6-HD at 5 min) and 0.214 (2,5-HD vs. 1,6-HD at 5 min). *p* values (**c**): 4.09977E-5 (1,4-BD vs. 2,5-HD), 0 (1,4-BD vs. 1,6-HD), and 0.00553 (2,5-HD vs. 1,6-HD).

The properties of the vesicles clusters was consistent with their liquid nature and their formation by LLPS principles. Thus, they had round shape and sharp boundaries (Fig. 1), and often fused with each other and coalesced into larger droplets (Fig. 3a). In samples incubated with CTX-HRP (Fig. 2), vesicles positive and negative for the endocytic tracer were randomly interspersed, indicating that they can enter the cluster and freely intermix, as observed for bona fide synaptic vesicles in presynaptic nerve terminals. Upon bleaching of mCherry-synapsin fluorescence, rapid recovery of fluorescence occurred (Fig. 3b,c), as in the case for synapsin droplets formed in vitro^7^. Furthermore, addition to cells of 3% 1,6-Hexanediol (1,6-HD), an alcohol known to disperse a variety of liquid biomolecular condensates via a mechanism that involves at least to some extent its hydrophobicity^15,16^, induced a reversible dispersion of both mCherry-synapsin and of synaptophysin immunoreactivity (Fig. 3d–f). 2,5-Hexanediol (2,5-HD) and 1,4-Butanediol (1,4-BD), two aliphatic alcohols with lower hydrophobicity^15,16^ were correspondingly less effective in dispersing the synaptophysin-positive and synapsin-positive droplets (Fig. 4).

We also wished to assess the dynamics of synaptophysin by live microscopy in COS7 cells. To this aim we expressed synaptophysin-EGFP in COS7 cells, as we could not use immunofluorescence. Surprisingly, even synaptophysin-EGFP alone, when expressed at very high levels, formed droplets with liquid-like properties, as shown by their property to fuse with each other (Supplementary Fig. 2a, b). CLEM confirmed that these structures were large clusters of small vesicles (Supplementary Fig. 2c) similar to those formed by synaptophysin and synapsin (Fig. 1b). Furthermore, synaptophysin-EGFP, when expressed at high levels, promoted formation of large clusters of small vesicles at non physiological locations even in cultured neurons (perikaryal and large dendrites), although it also accumulated in clusters of SVs in nerve terminals as expected (Supplementary Fig. 2d–g). The EGFP tag may induce clustering via its known property to undergo low affinity dimerization^17^. Since synaptophysin forms hexamers and each vesicle may contain multiple such hexamers^18,19^, each vesicle could act a multivalent particle that engages in multiple EGFP-mediated dimeric interactions with neighboring vesicles. Supporting this possibility, replacement of EGFP with monomeric EGFP at the C-terminus of synaptophysin abolished its property to form clusters (Supplementary Fig. 3). Importantly, however, as assessed by quantifying the levels of the mCherry and EGFP signal, co-expression of mCherry-synapsin and synaptophysin-EGFP lowered the concentration of synaptophysin-EGFP at which droplets started to appear (note that no mCherry-synapsin droplets occurred in the absence of synaptophysin irrespective of the concentration of synapsin) (Supplementary Fig. 4).

**Fig. 5:**
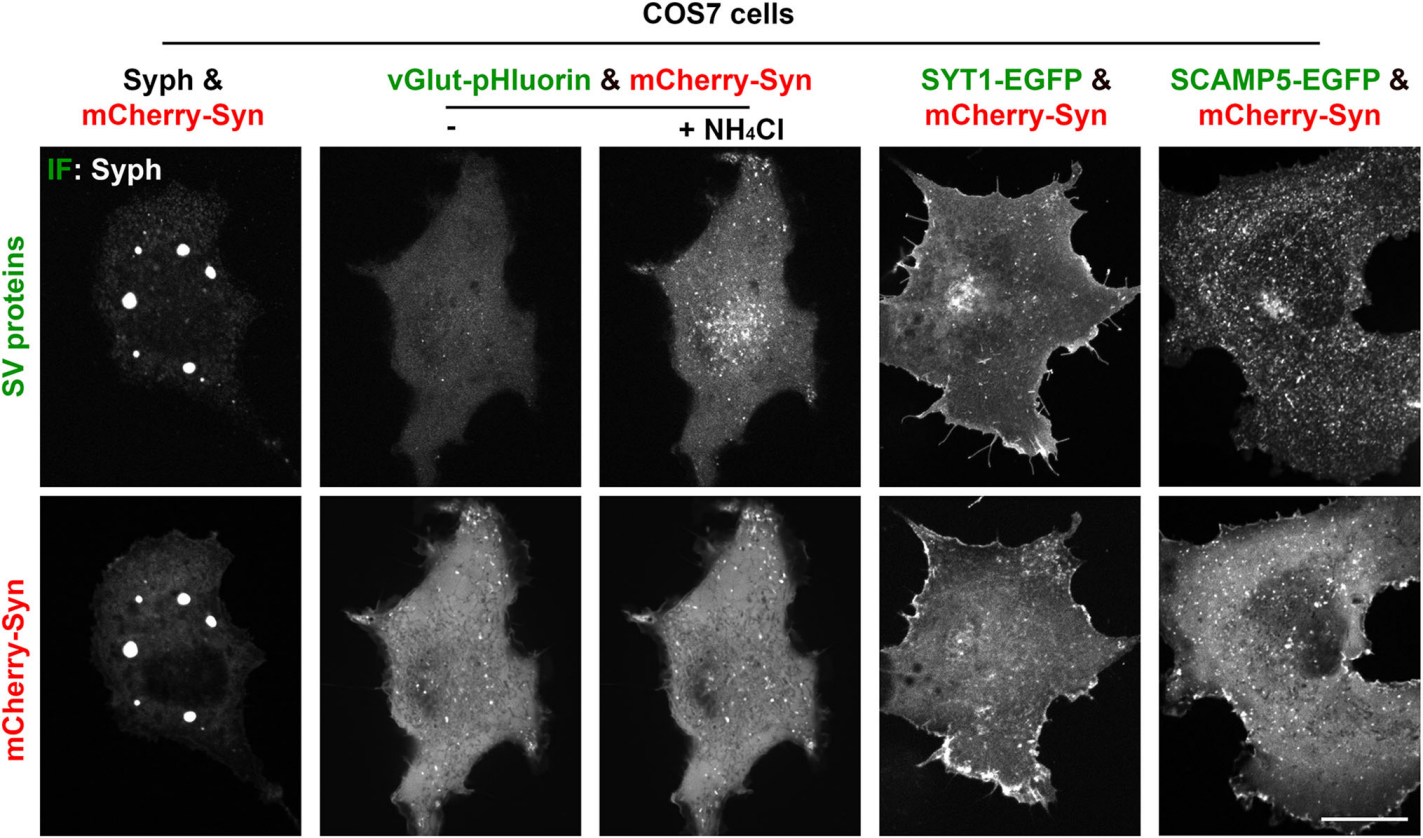
Synapsin does not form droplets when it is expressed with other integral SV proteins in fibroblasts. COS7 cells were transfected with mCherry-synapsin and with synaptophysin, vesicular glutamate transporter (vGlut)-pHluorin, synaptotagmin I (SYTI)-EGFP or secretory carrier associated membrane protein 5 (SCAMP5)-EGFP. Synaptophysin was visualized by immunofluorescence and vGlut-pHluorin by alkalinization with NH_4_Cl to allow detection of the entire pool of this protein, including the pool localized on acidified vesicles. Note that only mCherry-synapsin and synaptophysin expressing cells (left) show droplets. Scale bar = 20 μm.

The effects observed with synapsin had specificity, as synapsin did not form droplets when co-expressed in COS7 cells with other integral SV membrane proteins such as vesicular glutamate transporter 1^20–22^, synaptotagmin 1 (SYT1)^23,24^ or secretory carrier membrane protein 5 (SCAMP5)^25^ (Fig. 5).

### Electrostatic interactions between synapsin and the cytosolic domain of synaptophysin participate in phase separation

**Fig. 6:**
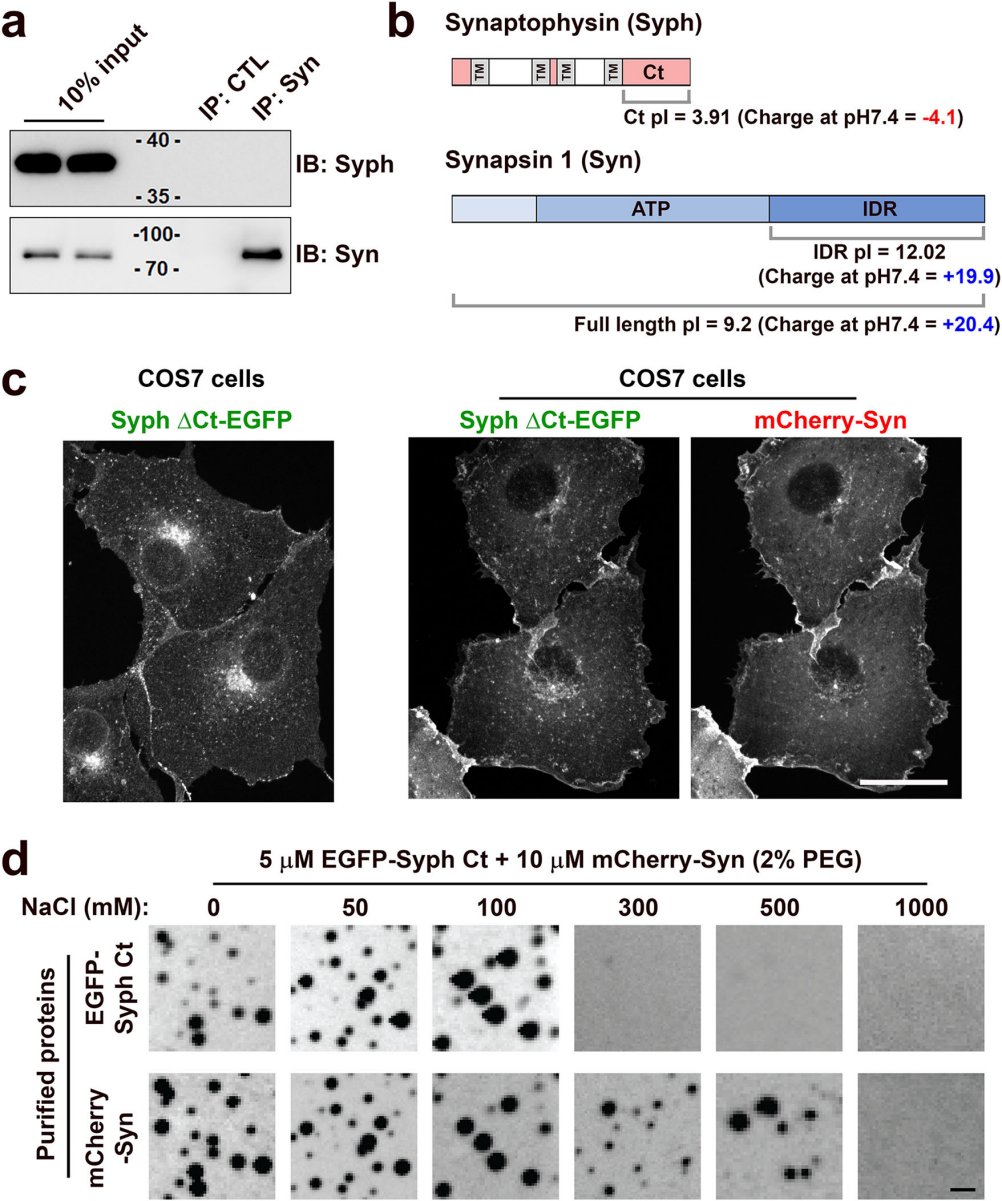
Electrostatic interaction between synapsin and the cytosolic domain of synaptophysin (Syph Ct) contribute to the clustering of the vesicles via phase separation. **a** Western blots of starting lysates and pellets of immunoprecipitations (IP) from DIV15 cultured hippocampal neurons. Anti-synaptophysin or anti-synapsin antibodies were used for immunoblotting (IB). **b** Domain structures of synaptophysin and synapsin and pIs (isoelectric points) values of *human* and *mouse* Syph Ct and human full-length synapsin. **c** EGFP-tagged C-terminal deletion mutant of synaptophysin (Syph ΔCt-EGFP, 1-218 a.a) was expressed alone or with mCherry-synapsin. **d** Co-assembly of the two purified proteins in vitro. 5 μM EGFP-Syph Ct and 10 μM mCherry-synapsin were mixed in a buffer containing 25 mM Tris-HCl (pH 7.4), 0.5 mM TCEP, 2% PEG and supplemented with NaCl of various concentrations (from 0 to 1 M). Increasing the concentration of NaCl results first in the dissociation of Syph Ct and then also in the dispersion of synapsin. Scale bars, **c** = 20 μm, **d** = 1 μm.

The striking synergistic effect of synaptophysin and synapsin in the formation of vesicle clusters suggested that these two proteins directly bind to each other. At least some such interactions must occur in trans, between synaptophysin on one vesicle and synapsin anchored to an adjacent vesicle via its bilayer binding properties. However, we found no evidence of a detectable physical interaction between them by immunoprecipitation (Fig. 6a), indicating that their interaction, direct or indirect, must be of low affinity but sufficient to drive condensate formation by multivalency. To further explore whether synapsin and synaptophysin alone can drive condensate formation, we performed cell-free assays using purified synapsin and the purified cytosolic C-terminal region of synaptophysin (Syph Ct), which is the main cytosolically exposed region of this transmembrane protein^26^ (Fig. 6b). The importance of this portion of synaptophysin in the co-assembly with synapsin was confirmed by the observation that a synaptophysin construct lacking this region (Syph ΔCt-EGFP) failed to co-assemble with synapsin in living cells (Fig. 6c).

Syph Ct, which is well-conserved among mammals, is negatively charged (pI = 3.91 (Ct only) in humans and mice)^26^ (Fig. 6b and Supplementary Fig. 5a). Conversely, synapsin has a polybasic C-terminal intrinsically disordered region (IDR) (pI = 12.02 (IDR) or 9.2 (full length) in humans) (Fig. 6b). Thus, synapsin may interact with Syph Ct through electrostatic interactions. We had previously shown that purified synapsin can phase separate and self-assemble into droplets either in the absence or presence of a crowding agent (2% PEG), but addition of PEG drastically accelerates droplet formation^7^. In contrast, purified EGFP-Syph Ct alone did not form droplets even in the presence of 2% PEG (Supplementary Fig. 5b). However, when co-incubated with synapsin (molar ratio synapsin-to-Syph Ct = 2-to-1) and in a buffer with salt concentration in the physiological range supplemented with 2% PEG, Syph Ct rapidly (less than 1 min) co-assembled with synapsin into droplets (Fig. 6d). In the absence of PEG, both proteins failed to form droplets within 5 min (Supplementary Fig. 5c). Raising the salt concentration of the medium to 300 mM NaCl, a condition known to be compatible with the occurrence of synapsin droplets^7^, rapidly dispersed EGFP-Syph Ct from the mCherry-synapsin condensates (Fig. 6d). This indicates the occurrence of a direct low affinity binding of synapsin to synaptophysin which is dependent, at least in part, on ionic interactions and which is weaker than homophilic interactions of synapsin. A further increase of the ionic strength (1 M NaCl) led to dispersion also of synapsin, as previously reported^7^ (Fig. 6d). The physiological significance of these finding is supported by the observation that even during the purification of SVs the retention of synapsin on the vesicle surface is critically dependent on maintaining a low ionic strength^27^. In contrast to these findings with purified proteins, Syph Ct-EGFP and mCherry-synapsin did not form droplets when expressed in COS7 cells (Supplementary Fig. 5d). Most likely, when Syph Ct is part of full length synaptophysin its propensity to participate in condensates formation is enhanced by multivalency as synaptophysin is an hexameric protein and each vesicle contains multiple copies of synaptophysin and thus exposes to its surface multiple copies of Syph Ct.

### CaMKII activation disperses vesicle clusters

**Fig. 7:**
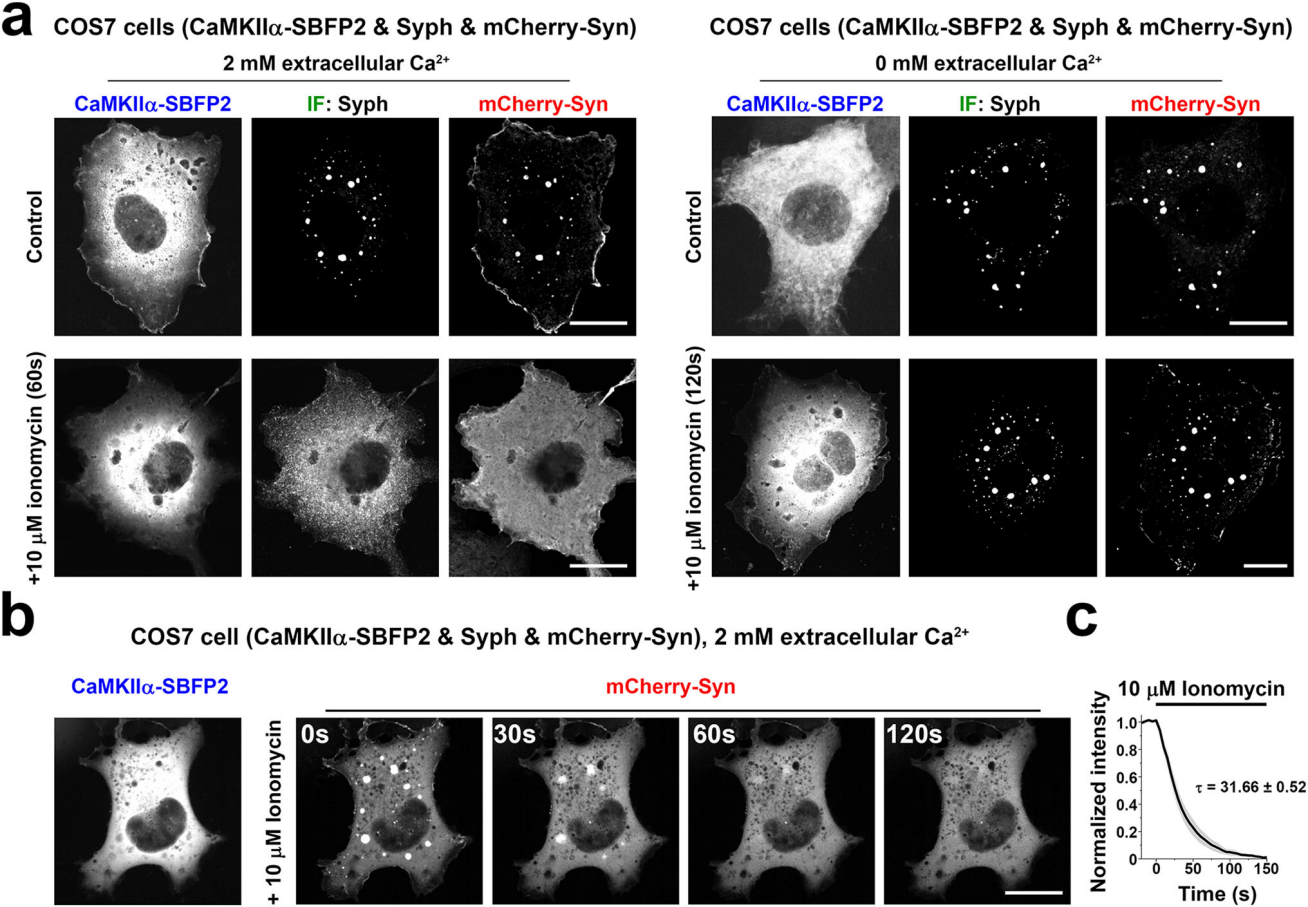
Activation of CaMKII dissociates synaptophysin and synapsin droplets. **a**–**c** COS7 cells were co-transfected with CaMKIIα-SBFP2, synaptophysin, and mCherry-synapsin. **a** Transfected cells were incubated with either vehicle or 10 μM ionomycin for 60 s in tyrode buffer containing either 2 mM Ca^2+^ (left) or 0 mM Ca^2+^ (right) and then immediately fixed. Synaptophysin was visualized by immunofluorescence (IF). **b** Representative time-lapse images of mCherry-synapsin after ionomycin treatment. **c** Analysis of the fluorescence intensities of mCherry-synapsin droplets after ionomycin addition. The decay time constant (τ) was obtained by a single exponential fit after ionomycin treatment. Values are means ± SEM. 61 droplets from 5 independent experiments were used for the analysis. Scale bars, **a** = left top: 20 μm, left bottom: 20 μm, right top: 15 μm and right bottom: 20 μm. **b** = 20 μm.

In vivo and in vitro interactions dependent on the basic IDR of synapsin are antagonized by its phosphorylation on site 2 and 3 by calcium/calmodulin-dependent protein kinase II (CaMKII), a kinase that binds to synapsin and acts physiologically to induce synapsin dissociation from SVs upon Ca^2+^-dependent stimulation^7,28^. To assess whether also the binding of synapsin to synaptophysin-positive vesicles in fibroblasts is regulated by this kinase, consistent with a charge-based interaction, we coexpressed in COS7 cells CaMKIIα-SBFP2, synaptophysin (untagged) and mCherry-synapsin. Addition of ionomycin to raise cytosolic Ca^2+^ and thus to activate CaMKII, induced a rapid and complete dispersion of synapsin/synaptophysin droplets, as shown by the dispersion of mCherry-synapsin (Fig. 7a left, Fig. 7b and c) and by the corresponding dispersion of synaptophysin, as shown by immunofluorescence (Fig. 7a, left). No effect on the droplets was observed in the absence of extracellular Ca^2+^ (Fig. 7a, right) or without CaMKII coexpression (Supplementary Fig. 6), confirming the Ca^2+^ dependency of synapsin and synaptophysin condensates.

## Discussion

Collectively, our results show that expression of only two presynaptic proteins in the cytoplasm of non-neuronal cells—the intrinsic membrane protein synaptophysin and the peripheral protein synapsin—is sufficient to assemble clusters of vesicles highly reminiscent of bona fide SV clusters in morphology and liquid properties. The small size of the vesicles in the clusters—in the same size range of SVs—is a particularly striking feature of the condensates. Like SVs, such vesicles are part of the endosomal system as they can be labeled by endocytic tracers. Whether synaptophysin helps generate them or whether it is simply a cargo of such vesicle remains a question for future studies. Our results also reveal that a multiplicity of low affinity interactions is critical for the assembly of the vesicles clusters, as typical of condensates generated by LLPS^3^. In fact, as our results with synaptophysin-EGFP show, the role of synapsin as a cross-linker of synaptophysin-positive vesicles can be replaced by the dimerization properties of EGFP. The interaction of synapsin with synaptophysin appears to be primarily an ionic-based interaction that capitalizes on the highly acidic nature of the cytosolic C-terminal region of synaptophysin and the highly basic intrinsically disordered C-terminal region of synapsin.

Gene KO studies have shown that the lack of all three synapsins impairs, but does not completely abolish, the accumulation of SVs at synapses^7,9,11^ and that the absence of synaptophysin does not have an obvious effect on their formation^29^. These findings indicate that the function of these proteins, and more so of synaptophysin, must be redundant with that of other SV proteins. Yet, the minimal reconstitution system shown here promises to be a powerful model system for further investigating mechanisms in the assembly of presynaptic structures.

## Methods

### Plasmid DNA construction

The *mouse* synaptophysin-EGFP (Syph-EGFP) N1 construct was kindly provided by Dr. Jane Sullivan (University of Washington, Seattle, WA). A stop codon was inserted after the synaptophysin sequence (GenBank: CAA65084.1) to express synaptophysin without the EGFP tag (Syph). The C-terminal cytoplasmic region of synaptophysin (a.a. 219–308) was amplified by PCR and cloned into the pEGFP-N1 vector. The N-terminal 6xHis tagged C-terminal cytoplasmic region of synaptophysin in a pEGFP-C1 vector was used for the in vitro assays. The C-terminal deletion mutant of synaptophysin (1–218 aa) was amplified and cloned into pEGFP-N1 vector. *Human* EGFP-synapsin 1 (EGFP-Syn) was subcloned into the pEGFP-C1 vector^7^. *Human* TOM20 was amplified from a HEK293T cDNA library by PCR and inserted into the EGFP-C1 vector. *Mouse* CaMKIIα was amplified from CaMKIIα-Venus from Addgene (plasmid 29427) and subcloned into the SBFP2-N1 vector. Each construct was validated by DNA sequencing.

### Antibodies

Antibodies used for western blotting: anti-synaptophysin (101 011, Synaptic Systems, Gottingen, Germany) and anti-synapsin (106 103, Synaptic Systems). Antibodies used for immunofluorescence: anti-synaptophysin (101 002, Synaptic Systems) and anti-synapsin (G246, kind gift of Reinhard Jahn).

### Cell culture and transfection

COS7 cells were maintained at 37 °C and 5% CO_2_ in Dulbecco’s Modified Eagle’s Medium (DMEM) supplemented with 10% FBS and 100 U/ml penicillin and 100 mg/ml streptomycin. Cells were transfected by Lipofectamine-2000 in a 35 mm MatTek dish (~70% confluency) with a total of 1.5 μg of plasmid DNA. Plasmid DNA was added to 100 μl opti-MEM and then mixed with 4 μl lipofectamine. After 20 min incubation at RT, the mixture was added to cells in serum/antibiotics free medium (DMEM) for 3 h at 37 °C in a CO_2_ incubator. The medium was replaced by growth medium and cells were further incubated for 18–36 h before imaging. Hippocampal neurons were cultured, transfected at 8 days in vitro using a calcium phosphate transfection method as previously described^30^, and fixed at 18 days in vitro. Briefly, hippocampus was dissected out from postnatal day 0 (P0) mouse brains and placed in ice-cold HBSS. Tissues were then digested for 20 min in a HBSS solution containing papain (20 U/ml) and DNase (20 μg/ml) at 37 °C. After trituration, dissociated cells were plated onto poly-d-lysine-coated coverslips to a density of 12,000–20,000 cells/cm^2^. Three hours after plating, the plating medium was exchanged to neurobasal medium containing 2% B-27 and 0.5 mM L-glutamine. Cells were maintained at 37 °C in a 5% CO_2_ humidified incubator. 30% of cultured medium was replaced with new complete neurobasal medium at DIV4, 7 and 14. For calcium phosphate transfection, 6 μg of plasmid DNA and 9.3 μl of 2 M CaCl_2_ were gently mixed with distilled water (Final volume: 75 μl) and added to the equal volume of 2× BBS (50 mM BES, 280 mM NaCl, and 1.5 mM Na_2_HPO_4_, pH 7.1). The mixture was incubated for 20 min at RT and added to neurons in transfection medium (1 mM pyruvate, 0.6% glucose, 2 mM glutamine, and 10 mM HEPES in MEM, pH 7.65) for 1 h at 37 °C and then the medium was replaced with wash medium (1 mM pyruvate, 0.6% glucose, 2 mM glutamine, and 10 mM HEPES in MEM, pH 7.35). After 30 min, the medium was again changed to the original medium. All adult mice for breeding were maintained on a 12 h light/dark cycle with standard mouse chow and water ad libitum. All animal experiments were approved by the Institutional Animal Care and Use Committees of Seoul National University and Yale University.

### Immunoprecipitation and western blotting

Cultured neurons were lysed with 1% triton X-100 lysis buffer (20 mM Tris-HCl, pH 8, 1% triton X-100, 10% glycerol, 137 mM NaCl, 2 mM EDTA, 1 mM PMSF, 10 mM leupeptin, 1.5 mM pepstatin, and 1 mM aprotinin). Lysates were centrifuged at 14,000×*g* for 20 min at 4 °C, the supernatants were collected and, following protein determination by the BCA assay kit (Thermo Scientific, MA), samples were processed by SDS-PAGE and transferred to PVDF membranes (Pall Life Sciences, Ann Arbor, MI). PVDF membranes were blocked with 5% skim milk in PBS and then incubated with primary antibodies (1:2000) followed by horseradish peroxidase (HRP)-conjugated secondary antibodies (1:5000) (Jackson Immuno Research Laboratories, West Grove, PA) which were detected by ECL (enhanced chemiluminescence reagent (AbClon, Seoul, South Korea)). For immunoprecipitation, lysates (500 μg) were incubated for 90 min at 4 °C with primary antibodies (1:500) and then, after a wash, for one additional hour at 4 °C with Protein A-Sepharose beads (GE healthcare). Beads were washed by lysis buffer, eluted with sample buffer and boiling (5 min), and the eluates were processed for SDS-PAGE and western blotting.

### Correlative light and electron microscopy (CLEM)

COS7 cells or cultured neurons were plated on 35 mm gridded, glass-bottom MatTek dish (P35G-1.5-14-CGRD) and transfected. Cells with/without CTX-HRP (10 μg/ml, Invitrogen, C34780) incubation were fixed with 4% PFA in PB and washed in PB. Regions of interest were selected by fluorescence microscopy and their coordinates on the grid were identified using phase contrast. Cells were further fixed with 2.5% glutaraldehyde in 0.1 M sodium cacodylate buffer, postfixed in 2% OsO_4_ and 1.5% K_4_Fe(CN)_6_ (Sigma-Aldrich, St. Louis, MO) in 0.1 M sodium cacodylate buffer, en bloc stained with 2% aqueous uranyl acetate, dehydrated, and embedded in Embed 812. Regions of interest were relocated based on the pre-recorded coordinates, sectioned and imaged. The HRP reaction was carried out with diaminobenzidene (Sigma-Aldrich, D-5637) (0.5 mg/ml) and H_2_O_2_ (JTBaker, 2186-01) (0.01%) in 0.1 M ammonium phosphate buffer (pH7.4) after the glutaraldehyde fixation step. For EM of synapses in situ, mice were anesthetized, perfused with 2% formaledehyde and 2.5% glutaraldehyde in 0.1 M sodium cacodylate buffer at 37 °C. Small pieces of cerebral cortex tissue were kept in the same fixative solution for another 2 h at room temperature, post-fixed in 2% OsO_4_ + 1.5% K_4_Fe(CN)_6_ (Sigma-Aldrich) in 0.1 M sodium cacodylate buffer for 1 h. Samples were subsequently stained with 2% aqueous uranyl acetate (1 h at RT), dehydrated in increasing concentration of ethanol and embedded in Embed 812. Ultrathin sections (50-60 nm) were examined in a Philips CM10 microscope at 80 kV and images were taken with iTEM (Soft Imaging System GmbH) and a Morada 1kx1k CCD camera (Olympus). Except when noted, all EM experiment components are from EMS, Hatfield, PA.

### Fluorescence live imaging

Spinning disk confocal (SDC) live microscopy was carried out at 35 °C either on a Nikon Ti-E inverted microscope using the Improvision UltraVIEW VoX system (PerkinElmer) and a planar Apo objective ×60, 1.49-NA. Fluorescence was detected by an EM‐CCD camera (C9100‐50; Hamamatsu Photonics). During imaging, cells were incubated in tyrode solution (136 mM NaCl, 2.5 mM KCl, 2 mM CaCl_2_, 1.3 mM MgCl_2_, 10 mM HEPES and 10 mM glucose). All images were analyzed with ImageJ.

#### FRAP

Time-lapse images were acquired every 2 s. After two images were acquired, a droplet was bleached by scanning with a 488 nm laser for 1 s and fluorescence recovery was subsequently imaged at 2 s intervals.

#### Aliphatic alcohol treatments

Synaptophysin and mCherry-synapsin expressing COS7 cells were briefly washed in pre-warmed tyrode, incubated with 3% alcohols [1,6-Hexanediol (Sigma-Aldrich, 240117), 1,4-Butanediol (Sigma-Aldrich, 493732) or 2,5-Hexanediol (Sigma-Aldrich, H11904)] in tyrode for various times.

#### Ionomycin

COS7 cells transfected with synaptophysin, mCherry-synapsin and with or without CaMKIIα-SBFP2, were briefly washed in a pre-warmed tyrode and then incubated with 10 µM ionomycin.

### Immunofluorescence

Cells were fixed with 4% PFA in 4% sucrose-containing 0.1 M phosphate buffer (pH7.3) (PB) for 15 min at RT and washed by PBS. Then, cells were incubated with primary antibodies diluted (1:1000) in blocking buffer (3% BSA, 0.2% Triton X-100 in PBS) for 1 h at RT, washed with PBS, incubated with Alexa Fluor-conjugated secondary antibodies (1:1000) in blocking buffer for 45 min at RT and finally washed again and mounted on coverslips.

### Quantification of droplet formation

COS7 cells were transfected with synaptophysin-EGFP and mCherry-synapsin, and fixed after 48 h after transfection. Imaging was performed using an Olympus IX-71 microscope and a ×40 objective lens (N.A. 1.0) with an Andor Sona sCMOS Camera driven by MetaMorph Imaging software (Molecular Devices). The average fluorescence intensity (intensity/cell area) values (reflecting the expression level of the fluorescent protein) from each cell were measured using MetaMorph and ImageJ. Presence of droplets was assessed as follows: Using ImageJ, we subtracted the background from each image and applied the threshold function to acquire a binarized image. Then, the number of droplets were quantified using the ‘Analyze Particles’ command. Droplets with a size smaller than 1 μm^2^ and with a circularity outside the 0.7 to 1 range were ignored [circularity = 4 pi (area/perimeter^2^); the value of 1.0 indicates a perfect circle]. If the droplet count was less than 5, we then visually examined the original image to ensure the presence of droplets. The analysis was performed at least three times on a single-blind basis to ensure reproducibility.

### Protein purification and cell-free assay

Synapsin (a.a. 1–705, human sequence) and Syph Ct (a.a. 219–308, mouse sequence) were expressed in Expi293 cells (Thermo Fisher Scientific) for three days following induction^7^. Cells were harvested and lysed in a buffer that contained 25 mM Tris-HCl (pH 7.4), 300 mM NaCl, 0.5 mM TCEP (buffer A), and protease inhibitor (Complete EDTA-free, Roche). The lysates were centrifuged for 1 h at 17,000×*g*, followed by a two-step purification. The first step was affinity purification on a Ni-NTA column (Clontech) for 45 min (elution with 400 mM Imidazole in buffer A). The second step was gel filtration chromatography (Superdex 200, GE Healthcare) in 25 mM Tris-HCl (pH 7.4), 150 mM NaCl, 0.5 mM TCEP (buffer B). For imaging, the two proteins were desalted using PD-10 column (Sigma-Aldrich), resuspended in 25 mM Tris-HCl (pH 7.4), 0.5 mM TCEP and supplemented with NaCl of various concentrations (from 0 to 1 M). The mixture was pipetted on 35‐mm glass-bottom dishes (MatTek Corp). When formed, droplets of synapsin with Syph Ct adhered to the glass surface that was coated with poly-D-lysine (Sigma-Aldrich). Imaging was performed as described above for live imaging, but at RT.

### Statistics and reproducibility

Normality of data was confirmed by using the Kolmogorov–Smirnov normality test. After the assumption was validated, the Student’s two-sample t-test was used to compare two independent groups, while ANOVA followed by Tukey’s honest significant difference (HSD) post hoc test was applied for multiple comparisons. Sigma plot, Origin 9.0 and SPSS (IBM) software were used for statistical comparisons. The relevant p values are presented in the figure legends and, unless otherwise indicated, data are presented as means ± SEM or SD, with *n* indicating the number of independent experiments. All representative light microscopy images of cells were obtained from experiments which were repeated at least three times. CLEM experiments were repeated twice with similar results. The Western blot was repeated four times. The in vitro assay was performed three times and representative images are shown.

### Sequence alignment

Alignment of synaptophysin sequences from different species was performed by using UniProt, Clustal Omega, and Jalview.

## Supplementary information

Supplementary Information

## Data Availability

The data that support the findings of this study are available from the corresponding authors upon reasonable request. Sequence for the mouse synaptophysin has previously been deposited in NCBI (accession number: CAA65084.1) (https://www.ncbi.nlm.nih.gov/protein/CAA65084). Source data are provided with this paper.

## Source data

Source Data
