# Supplementary material for: Cooperative function of synaptophysin and synapsin in the generation of synaptic vesicle-like clusters in non-neuronal cells
Source: Nat Commun. 2021 Jan 11;12:263. doi: 10.1038/s41467-020-20462-z (PMC7801664; doi:10.1038/s41467-020-20462-z)
Supplement: Supplementary file 1 — Supplementary Information [file 41467_2020_20462_MOESM1_ESM.pdf]

Supplementary Information for

Cooperative function of Synaptophysin and Synapsin in the generation synaptic vesicle-like clusters in non-neuronal cells

Daehun Park[1], Yumei Wu[1,†], Sang-Eun Lee[2,†], Goeun Kim[2],
Seonyoung Jeong[2], Dragomir Milovanovic[1,3], Pietro De Camilli[1,*] and Sunghoe Chang[2,*]

[1]Departments of Neuroscience and Cell Biology, Howard Hughes Medical Institute, Program in Cellular Neuroscience, Neurodegeneration and Repair, Kavli Institute for Neuroscience, Yale University School of Medicine, New Haven, Connecticut 06510, USA
[2]Department of Physiology and Biomedical Sciences, Seoul National University College of Medicine, Seoul 03080, South Korea
[3]Laboratory of Molecular Neuroscience, German Center for Neurodegenerative Diseases (DZNE), Charitéplatz 1, 10117 Berlin, Germany

[†] These authors contributed equally

*Address correspondence to: pietro.decamilli@yale.edu, sunghoe@snu.ac.kr

This file includes:

Supplementary Fig.1 to 6

Supplementary Figures and legends

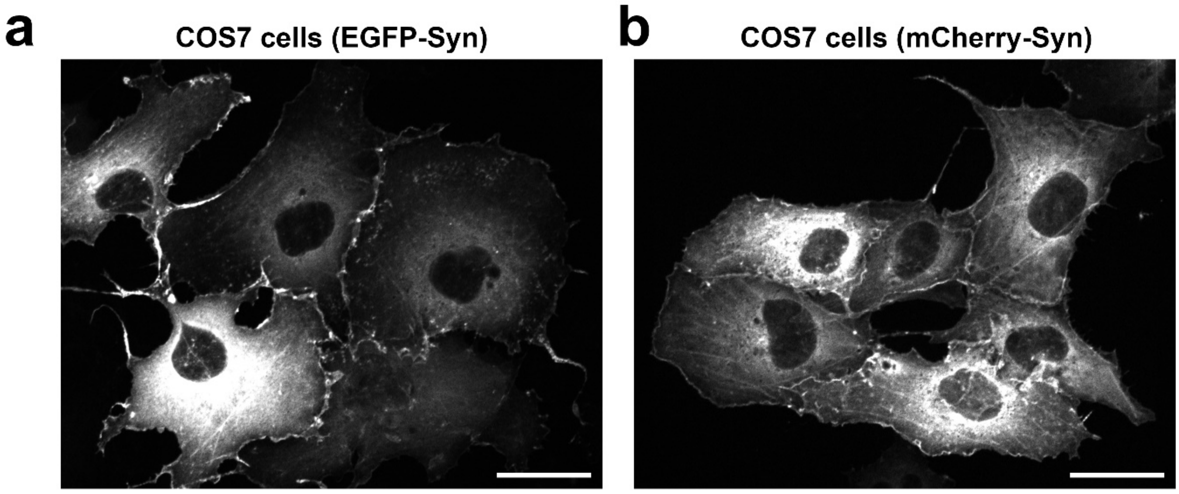

Supplementary Fig. 1. Synapsin does not form droplets when expressed alone in living cells. COS7 cells were transfected with either EGFP-synapsin **(a)** or mCherry-synapsin **(b)**. Scale bars = 20 µm.

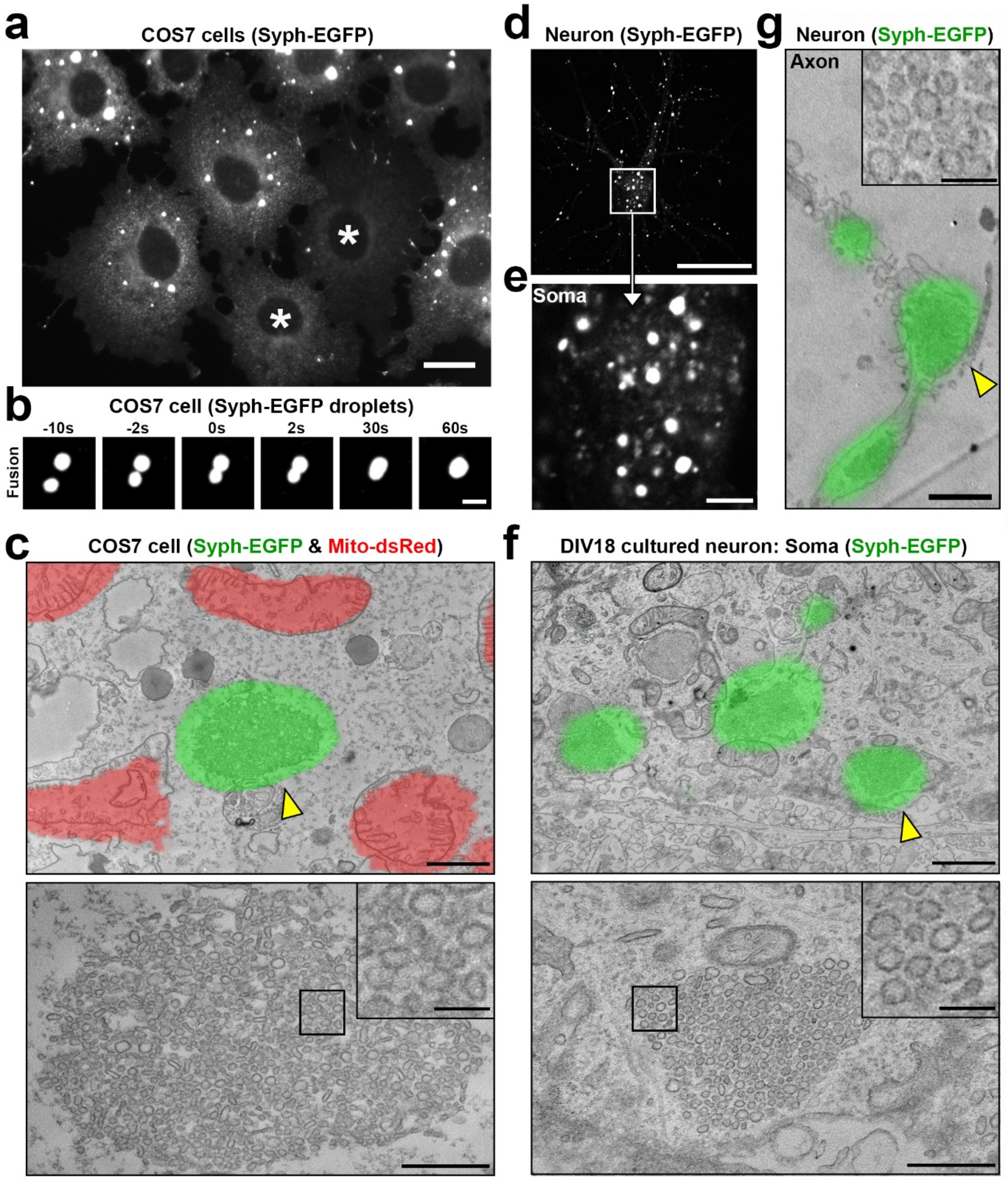

Supplementary Fig. 2. Presence of an EGFP tag at C-terminus of synaptophysin promotes it coalescence into LLPS droplets. a, COS7 cells that express high levels of synaptophysin-EGFP display synaptophysin droplets even without co-expression of synapsin. Note that low expressing cells (white asterisks) do not contain droplets. **b**, Fusion of two synaptophysin-EGFP droplets. **c**, CLEM of COS7 cells expressing synaptophysin-EGFP and Mito-dsRed, showing that a synaptophysin-EGFP positive droplets (top, arrowhead) is represented by a cluster of small vesicles (bottom). **d** and **e**, Cultured mouse hippocampal neuron expressing high levels of synaptophysin-EGFP showing EGFP-positive droplets in all cell compartments including the soma, shown at high magnification in **e**. **f**, CLEM images showing that these droplets (top) are indeed clusters of small vesicles (bottom). **g**, CLEM images of axons in synaptophysin-EGFP expressing cultured hippocampal neurons. The inset shows a detail of the cluster indicated by an arrowhead. Scale bars, **a** = 20 µm, **b** = 2 µm, **c** = 1 µm (top), 500 nm (bottom) and 100 nm (inset), **d** = 40 µm, **e** = 5 µm, **f** = 1 µm and 100 nm (Inset), **g** = 1 µm and 100 nm (Inset).

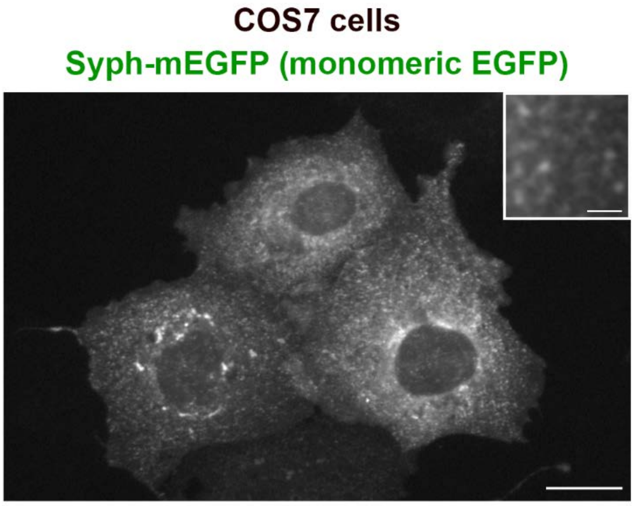

Supplementary Fig. 3. Synaptophysin with a monomeric EGFP (mEGFP) tag at its C-terminal does not induce synaptophysin droplet formation in cells. COS7 cells were transfected with Syph-mEGFP. Scale bar, 20 μm or 2 μm (inset).

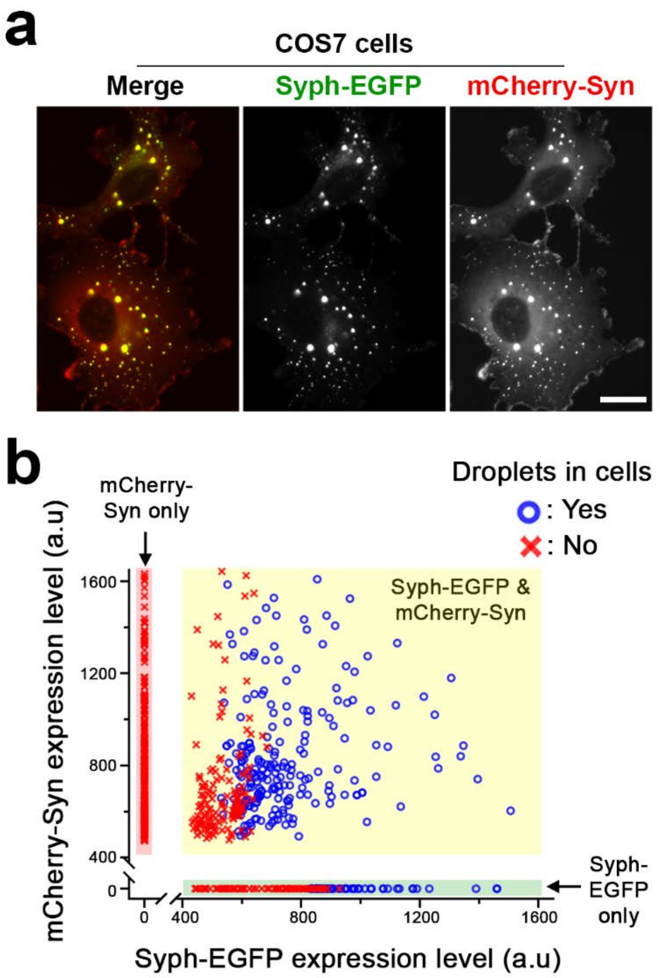

Supplementary Fig. 4. The propensity of synaptophysin-EGFP to form droplets when expressed alone is enhanced by co-expression of mCherry-synapsin. a, Synaptophysin-EGFP and mCherry-synapsin co-assemble in the same droplets when expressed in COS7 cells. **b**, COS7 cells were transfected with synaptophysin-EGFP alone, mCherry-synapsin alone, or both proteins together. Average expression levels of each protein in each cell (total fluorescent intensity/cell area) were measured and the position representing this value is indicated by blue circles or a red X depending on the presence or absence of droplets. Note that mCherry-synapsin alone failed to form droplets regardless of expression levels. A total of 775 cells was analyzed. Scale bar in **a** = 20 μm.

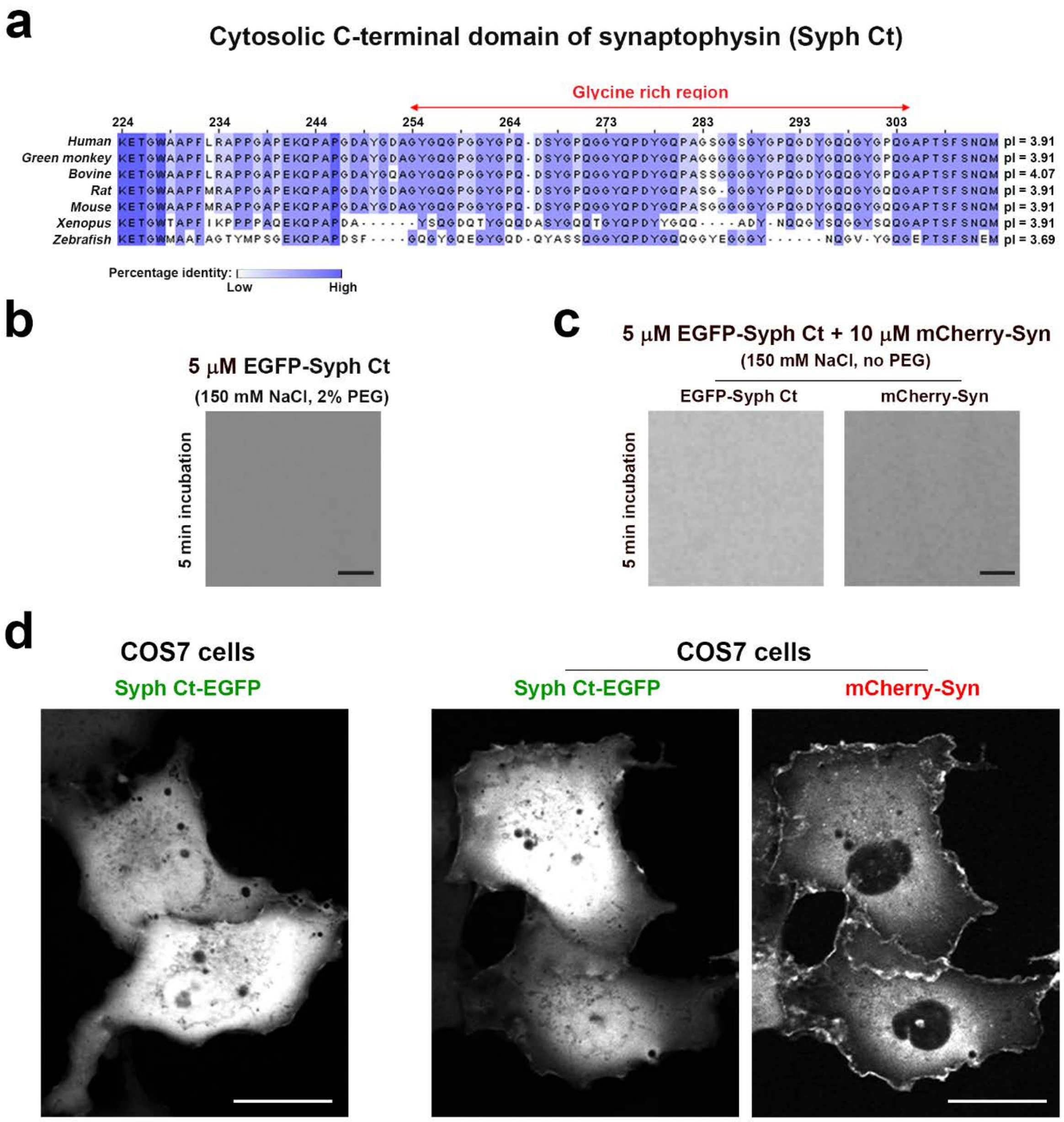

Supplementary Fig. 5. Properties of cytosolic C-terminal region of synaptophysin (Syph Ct). a, Sequence alignment of Syph Ct from different species. **b**, Purified EGFP-Syph Ct does not form droplets when incubated for 5 min in a buffer of physiological salt concentration (150 mM NaCl) with crowding reagent (2% PEG). **c**, Fluorescence images of mixture of purified EGFP-Syph Ct (5 μM) and mCherry-synapsin (10 μM) after 5 min incubation in a buffer of physiological salt concentration (150 mM NaCl) without PEG. **d**, COS7 cells were transfected as indicated and fixed before imaging. Note that Syph Ct-EGFP has a diffuse cytosolic distribution and does not make droplets even it is overexpressed with mCherry-synapsin. **b** = 1 μm, **c** = 1 μm, **d** = 20 μm.

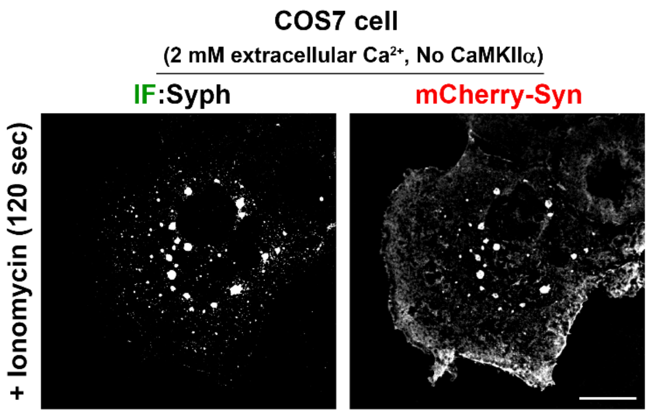

Supplementary Fig. 6. CaMKIIα expression is required for the dispersion of synapsin and synaptophysin droplets. COS7 cells co-expressing synaptophysin and mCherry-synapsin, but not CaMKIIα, were treated with 10 μM ionomycin in 2 mM Ca^{2+} containing tyrode buffer for 120 sec and fixed for immunofluorescence (IF). Scale bar = 20 μm.
